# The compartmental approach to revision of partial knee arthroplasty results in nearer-normal gait and improved patient reported outcomes compared to total knee arthroplasty

**DOI:** 10.1007/s00167-021-06691-9

**Published:** 2021-08-20

**Authors:** Amy J. Garner, Oliver W. Dandridge, Richard J. van Arkel, Justin P. Cobb

**Affiliations:** 1 Imperial College London, MSk Lab, Sir Michael Uren Biomedical Engineering Research Hub, Imperial College London, White City Campus, 80-92 Wood Lane, London, W12 0BZ UK; 2 Royal College of Surgeons of England and Dunhill Medical Trust Clinical Research Fellowship, Royal College of Surgeons of England, 35-43 Lincoln’s Inn Fields, London, WC2A 3PE UK; 3 Health Education Kent, Surrey and Sussex, Stewart House, 32 Russell Square, London, WC1B 5DN UK; 4 Biomechanics Group, Department of Mechanical Engineering, Imperial College London, City & Guilds Building, Exhibition Road, London, SW7 9AG UK

**Keywords:** Multicompartmental arthroplasty, Compartmental arthroplasty, Bicompartmental, Bi-unicondylar, Gait, Satisfaction

## Abstract

**Purpose:**

This study investigated the gait and patient reported outcome measures of subjects converted from a partial knee arthroplasty to combined partial knee arthroplasty, using a compartmental approach. Healthy subjects and primary total knee arthroplasty patients were used as control groups.

**Methods:**

Twenty-three patients converted from partial to combined partial knee arthroplasty were measured on the instrumented treadmill at top walking speeds, using standard gait metrics. Data were compared to healthy controls (*n* = 22) and primary posterior cruciate-retaining total knee arthroplasty subjects (*n* = 23) where surgery were performed for one or two-compartment osteoarthritis. Groups were matched for age, sex and body mass index. At the time of gait analysis, combined partial knee arthroplasty subjects were median 17 months post-revision surgery (range 4–81 months) while the total knee arthroplasty group was median 16 months post-surgery (range 6–150 months). Oxford Knee Scores and EuroQol-5D 5L scores were recorded at the time of treadmill assessment, and results analysed by question and domain.

**Results:**

Subjects revised from partial to combined partial knee arthroplasty walked 16% faster than total knee arthroplasty (mean top walking speed 6.4 ± 0.8 km/h, vs. 5.5 ± 0.7 km/h *p* = 0.003), demonstrating nearer-normal weight-acceptance rate (*p* < 0.001), maximum weight-acceptance force (*p* < 0.006), mid-stance force (*p* < 0.03), contact time (*p* < 0.02), double support time (*p* < 0.009), step length (*p* = 0.003) and stride length (*p* = 0.051) compared to primary total knee arthroplasty. Combined partial knee arthroplasty subjects had a median Oxford Knee Score of 43 (interquartile range 39–47) vs. 38 (interquartile range 32–41, *p* < 0.02) and reported a median EQ-5D 0.94 (interquartile range 0.87–1.0) vs. 0.84 (interquartile range 0.80–0.89, *p* = 0.006).

**Conclusion:**

This study finds that a compartmental approach to native compartment degeneration following partial knee arthroplasty results in nearer-normal gait and improved patient satisfaction compared to total knee arthroplasty.

**Level of evidence:**

III.

**Supplementary information:**

The online version contains supplementary material available at 10.1007/s00167-021-06691-9.

## Introduction

Partial knee arthroplasty (PKA) may be appropriate for up to 50% of primary knee arthroplasty cases [28] but currently accounts for just 10% of procedures in large joint replacement registries [1, 26]. In part, the low usage rate of PKA is attributed to the significantly higher revision rates compared to total knee arthroplasty (TKA) [1, 26], though just 17% of patients have tricompartmental disease [38]. At 13 years, cumulative revision rates of cemented unicompartmental arthroplasty (UKA) are 14.9%, while for primary patellofemoral arthroplasty (PFA) it is 24.4%, compared to 4.18% for the TKA over the same time frame [1].

**Fig. 1:**
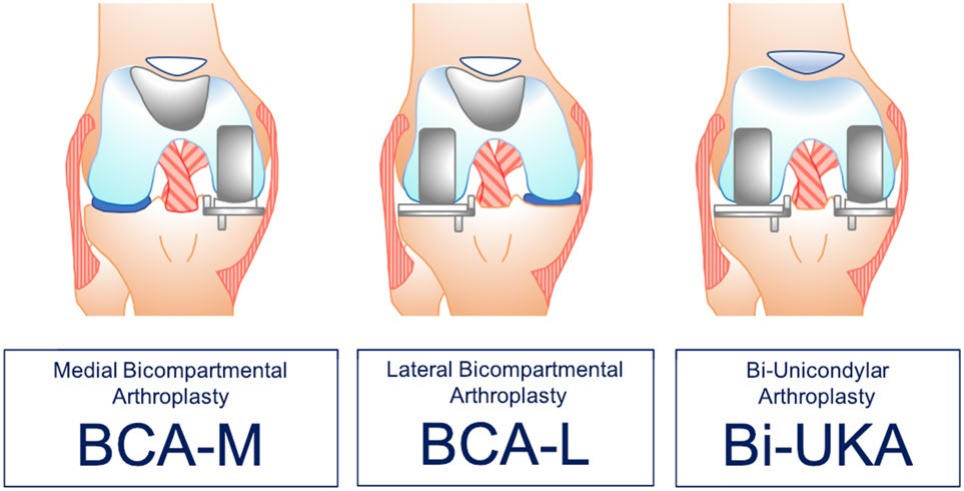
Classification of combined partial knee arthroplasty. Adapted from Garner et al. [14]

Progression of osteoarthritis remains the most common reason for revision of PKA [1, 22, 37] and most often involves the implantation of a standard primary TKA implant. In some instances, PKA removal can result in significant bone loss, necessitating the use of stems, metaphyseal augments or implants with increased constraint, potentially reducing post-revision patient satisfaction and function [22, 29, 37]. An alternative to revision to TKA is the addition of a second PKA to treat a newly symptomatic native compartment. This ‘compartmental approach’ converts PKA to a combined partial knee arthroplasty (CPKA) without the need for removal of a well-functioning primary PKA [18], with some evidence of successful outcomes in the medium [2, 7, 8] and longer term [19, 35]. CPKA have been classified according to the combination of PKA used and may be bicompartmental (BCA), that is the combination of PFA with either a medial (BCA-M) or lateral (BCA-L) UKA, or bi-unicondylar (Bi-UKA), the ipsilateral combination of medial and lateral UKA [14, 18] (Fig. 1.)

**Fig. 2:**
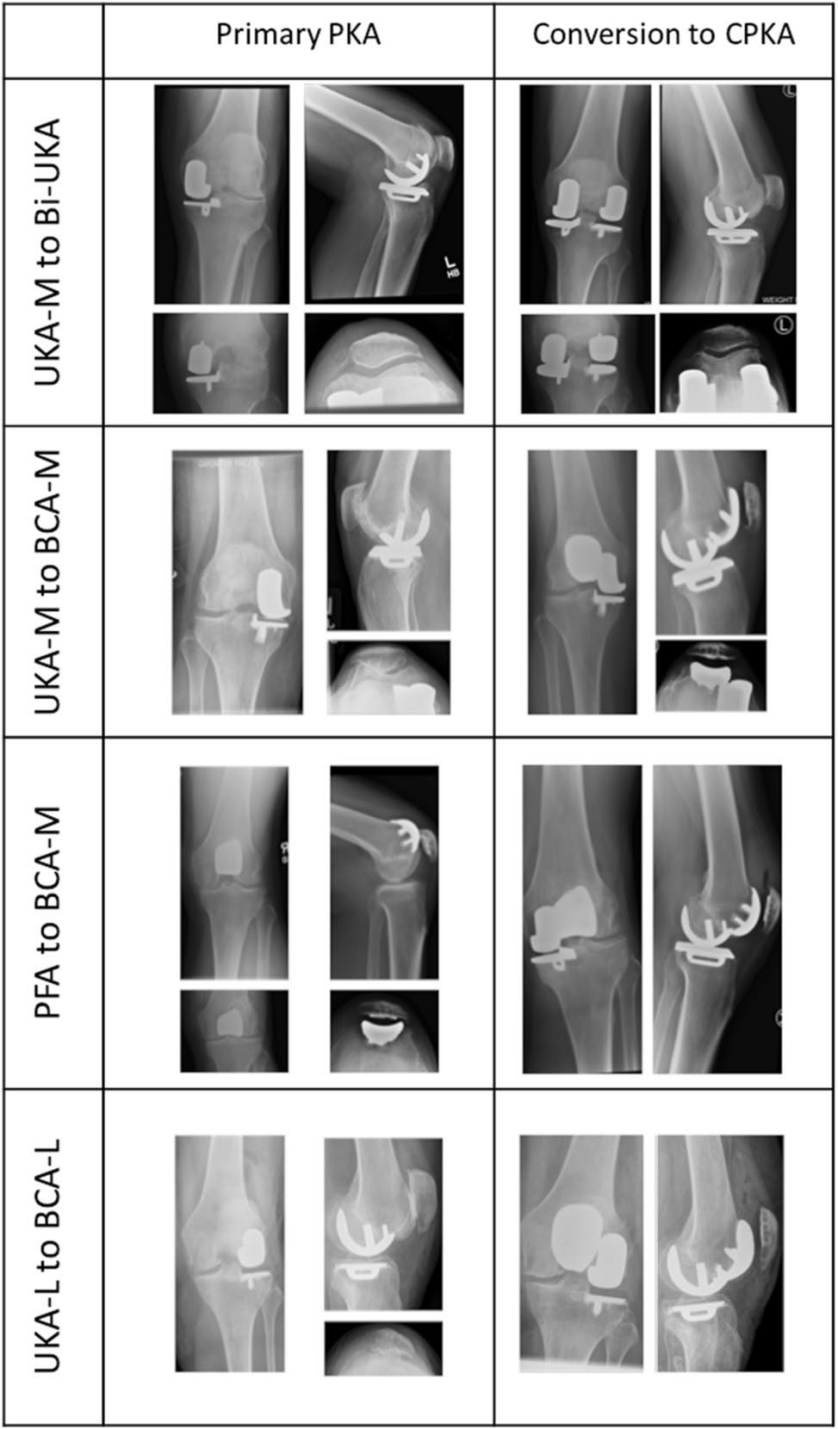
Radiographic examples of partial knee arthroplasty (PKA) procedures revised to combined partial knee arthroplasty (CPKA) for native compartment degeneration, using a compartmental approach. Medial unicompartmental arthroplasty (UKA-M), lateral unicompartmental arthroplasty (UKA-L), patellofemoral arthroplasty (PFA), medial bicompartmental arthroplasty (BCA-M), lateral bicompartmental arthroplasty (BCA-L), bi-unicondylar arthroplasty (Bi-UKA)

The functional outcomes and patient reported outcome metrics (PROMs) associated with CPKA in the staged setting (Fig. 2) are not known [12]. This study aimed to compare the post-operative gait and patient reported outcomes of staged CPKA to those of healthy controls and primary TKA subjects. The null hypothesis was that there would be no difference between staged CPKA and primary TKA in terms of gait and PROMs.

## Patients and methods

### The compartmental approach

Primary PKA had been performed for single compartment OA. The indications for conversion to CPKA are outlined in Table 1, primarily for degeneration of one of the remaining compartments, where the third compartment was intact and disease-free on plain radiographs, and the ACL functional on clinical examination and radiographic imaging (< 7 mm anterior tibial translation [9]). In the practise of the senior author, the absence of a functional ACL is a relative contraindication to conversion to CPKA, permissible in the elderly, where overall laxity of the joint is reduced, and the risk of major revision surgery from PKA to TKA outweighs the possibility of reduced anterior–posterior stability with CPKA. Inflammatory arthropathy is considered an absolute contraindication.

**Table 1:** Indications and contra-indications for conversion of PKA to CPKA

Indications for conversion to CPKA	Contra-indications
Osteoarthritic degeneration of a single native compartment	Osteoarthritic degeneration of two-native compartments
Well-functioning primary partial knee arthroplasty in situ^a^	Loose/unstable/problematic primary partial knee arthroplasty^a^
Functional anterior cruciate ligament^b^	Anterior cruciate ligament dysfunction^b^
Correctable varus/valgus	Inflammatory arthropathy
Medically high risk for revision surgery	Evidence of periprosthetic infection

^a^A worn polyethylene bearing may be exchanged at the time of second surgery and is not considered a contraindication

^b^ACL dysfunction in the elderly is a relative contraindication, provided that the knee is otherwise stable

Pre-operative imaging included plain radiographs of the knee, specifically the anterior–posterior, lateral, skyline and Rosenberg views. Use of cross-sectional imagine including magnetic resonance imaging was not routinely used. Procedures were performed using conventional instrumentation. Where conversion to BCA occurred, the original approach was re-used and extended as required. Where conversion to Bi-UKA occurred, if the original incision was in the midline, it was re-opened, and then a new parapatellar arthrotomy used to approach the native compartment (for example, a lateral parapatellar approach would be used to approach the lateral compartment, even though the original procedure used a medial parapatellar approach to the medial compartment). If the original incision was medial or lateral to the midline, a new, parallel incision was made over the native compartment. There were no reported incidences of unplanned intra-operative conversion to TKA.

### Gait analysis

**Fig. 3:**
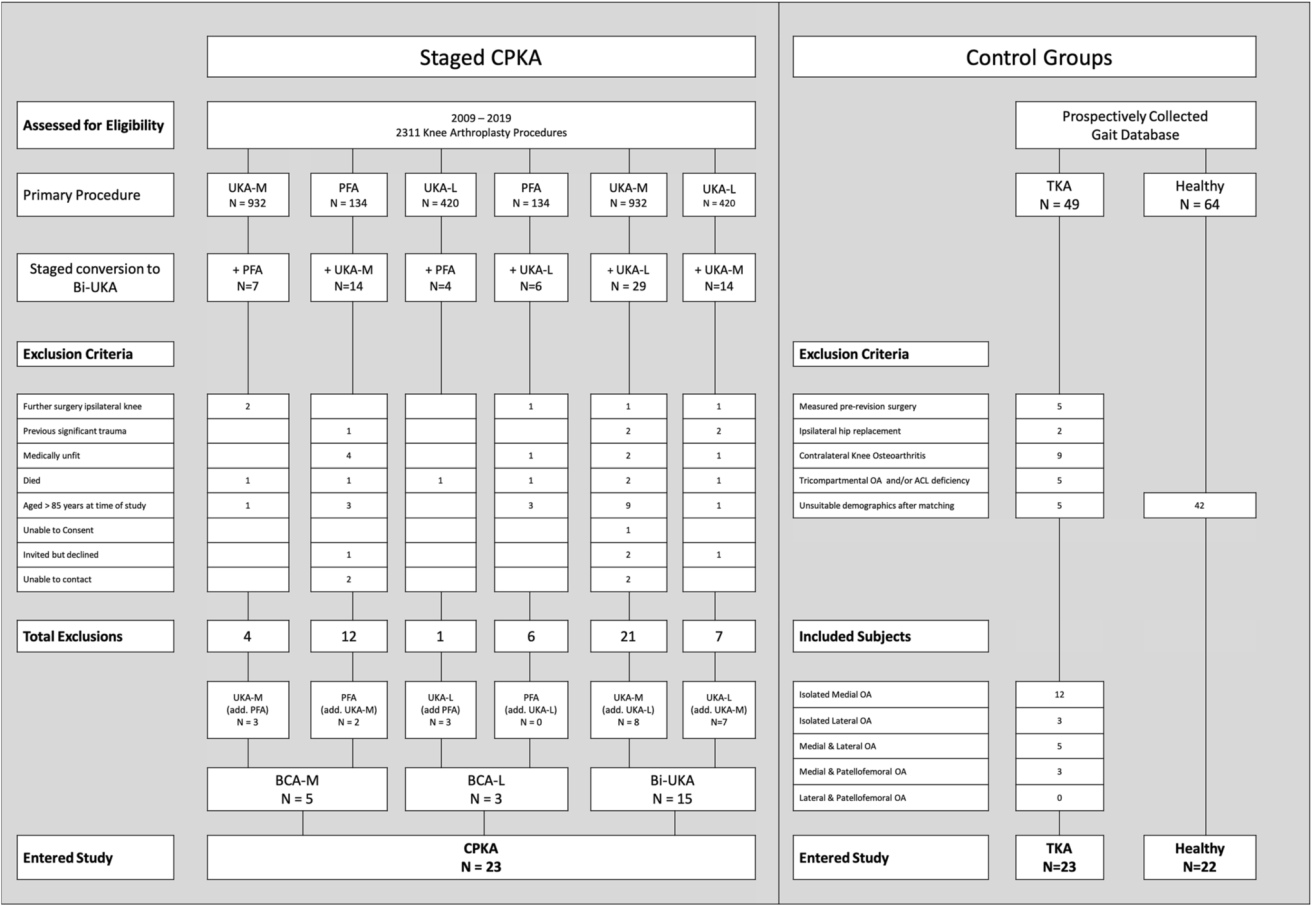
STROBE diagram depicting the route of subject entry into the study. Number of subjects (*N* =), partial knee arthroplasty (PKA), medial unicompartmental knee arthroplasty (UKA-M), lateral unicompartmental knee arthroplasty (UKA-L), patellofemoral arthroplasty (PKA), medial bicompartmental knee arthroplasty (BCA-M), lateral bicompartmental knee arthroplasty (BCA-L), bi-unicondylar knee arthroplasty (Bi-UKA), combined partial knee arthroplasty (CPKA), posterior cruciate-retaining total knee arthroplasty (TKA)

A retrospective cohort study was conducted, based on the operative caseload of the senior author, from 2009 to 2019. All subjects converted from a PKA to CPKA were considered for the study. Forty-six CPKA patients were excluded (Fig. 3, Supplementary Table A), three patients declined to participate following telephone invitation, none of whom reported further ipsilateral knee surgery. Four patients were uncontactable via telephone or e-mail. Twenty-three CPKA subjects entered the study (Fig. 3). They consisted of staged BCA-M (*n* = 5, 83% female), staged BCA-L (*n* = 3, 100% female) and staged Bi-UKA (*n* = 15, 27% female). Of them, eight had undergone primary PKA under the care of other surgeons, therefore, the combinations of implants varied (Supplementary Table B). Of the CPKA group, five subjects had undergone contralateral UKA-M and one had a contralateral UKA-L.

### Matching from the prospectively collected gait database

For 9 years, with informed consent and ethical approval (Imperial College Healthcare NHS Trust, 10/H0807/101 and NRES Committee South Central, 136430) three high-volume senior surgeons have contributed arthroplasty patients for measurement on the instrumented treadmill (Kistler Gaitway, Kistler Instrument Corporation, Amherst, NY). Subject data, in addition to that of healthy volunteers, has been pooled to form a prospectively collected gait database. Healthy controls (*n* = 22) were identified from this database, matched for age (*p* = 0.2), sex (*p* = 1), body mass index (*p* = 0.4) and height (*p* = 0.8) to those of the CPKA group (Table 2, Fig. 3.)

**Table 2:** Demographics, gait characteristics at top walking speeds and patient reported outcomes of staged combined partial knee arthroplasty (CPKA) subjects compared to healthy controls and total knee arthroplasty (TKA) subjects

Subject	Healthy	Staged CPKA	TKA
Number of knees (*N* =)	22	23	23
Sex: *M*:*F*	10:12	11:12	10:13
Age (years)	64 ± 8	68 ± 10	68 ± 10
Body mass index (kg/m^2^)	26.8 ± 5.4	28.4 ± 5.4	28.7 ± 3.5
Height (cm)	170 ± 9	172 ± 6	171 ± 11
Mean months post-surgery (SD)		22 ± 21^c^	42 ± 50^c^
Median months post-surgery (range)		17 (4–81)	16 (6–150)
Top walking speed (km/h)	7.2 ± 0.7^a,b^	6.4 ± 0.8^a,c^	5.5 ± 0.7^b,c^
Hof speed (H)	0.73 ± 0.1^a,b^	0.64 ± 0.1^a,c^	0.55 ± 0.1^b,c^
Weight acceptance rate (BW/s)	10.8 ± 3.5^b^	10.6 ± 3.6^c^	6.8 ± 2.6^b,c^
Maximum weight-acceptance force (BW)	1.6 ± 0.2^a,b^	1.4 ± 0.2^a,c^	1.2 ± 0.1^b,c^
Mid-stance force (BW)	0.5 ± 0.1^a,b^	0.6 ± 0.1^a,c^	0.7 ± 0.1^b,c^
Push-off force (BW)	0.98 ± 0.2	0.96 ± 0.1	0.97 ± 0.1
Push-off rate (BW/s)	4.1 ± 1.0	4.0 ± 0.9	3.6 ± 0.7
Step length (cm)	82 ± 8^b^	79 ± 7^c^	70 ± 7^b,c^
Stride length (cm)	165 ± 16^b^	156 ± 14	144 ± 17^b^
Gait width (cm)	12 ± 3	14 ± 2	13 ± 4
Cadence (step/min)	60 ± 5^b^	57 ± 5	53 ± 5^b^
Impulse (BW/s)	387 ± 23	378 ± 22	375 ± 21
Double support time (s)	0.28 ± 0.1^b^	0.29 ± 0.1^c^	0.36 ± 0.1^b,c^
Contact time (s)	1.3 ± 0.1^b^	1.4 ± 0.1^c^	1.5 ± 0.2^b,c^
OKS mean (range)		41.3 ± 7.4^c^	37.1 ± 5.1^c^
OKS median (interquartile range)		43 (39–47)	38 (32–41)
EQ-5D mean		0.91 ± 0.1^c^	0.83 ± 0.1^c^
EQ-5D median (interquartile range)		0.94 (0.87–1.0)	0.84 (0.80–0.89)

All values are means with standard deviations unless otherwise stated. Demongraphics subject to ANOVA, gait variables subject to Kruskal–Wallis then Mann–Whitney test with Bonferroni correction

*BW* normalised to body weight

All tests, significance *p* value < 0.05.

^a^Healthy vs. CPKA < 0.05

^b^Healthy vs. TKA *p* < 0.05

^c^CPKA vs. TKA *p* < 0.05

### Matching to TKA subjects

The design of this study was ‘non-inferiority’ to primary TKA. Forty-nine posterior cruciate-retaining TKA subjects were identified from the prospectively collected database (Fig. 3). TKA subjects were excluded if they were pre-revision (*n* = 5), had an ipsilateral hip replacement (*n* = 2), contralateral knee OA (*n* = 9) or unsuitable demographics after matching (*n* = 5). Of the remaining, pre-operative radiographs were assessed for Kellgren and Lawrence (KL) grade per compartment and anterior tibial translation > 7 mm, suggestive of ACL dysfunction [9]. TKA subjects (*n* = 5) were excluded if they had tricompartmental OA (KL ≥ 2) or radiographic evidence of anterior cruciate ligament (ACL) dysfunction. Twenty-three posterior cruciate-retaining TKA subjects entered this study, of whom 15 had KL ≥ 2 OA confined to the medial (*n* = 12) or lateral (*n* = 3) compartment, with the remaining 8 subjects having two-compartment disease (medial and lateral *n* = 5, medial and patellofemoral *n* = 3). TKA subjects were median 16 months post-surgery (range 6–150 months). All would have been eligible for PKA or CPKA under the senior author’s practice.

### Treadmill testing

With ethical approval from our institutional review board (Imperial College Healthcare NHS Trust 10/H0807/101 and NRES Committee South Central IRAS 136430) and informed consent, subjects were measured on the flat instrumented treadmill, by a research assistant blinded to the study group, following a 2 min acclimatisation ‘warm up’ at 3.5 km/h. The speed was then increased in 0.5 km/h increments, dictated by the subject, until they reached their ‘top walking speed’ defined as the fastest speed at which they could walk comfortably or before they felt compelled to run. All subjects completed the test without the assistance of the hand safety rail. The vertical component of the ground reaction forces; temporospatial measurements and centre of pressure readings for both limbs were recorded by two tandem force plates, sampling at 100 Hz frequency over 10 s, located beneath the moving belt. Data were normalised to account for differences in body mass (body mass/gravity) and leg length, using Hof scaling [20].

### Statistical analysis

The difference in gait of medial UKA subjects compared to TKA subjects at top walking speeds formed the basis of a power calculation, resolving that 16 subjects per group would be required to detect gait differences at top walking speeds, with 80% power and 95% confidence [23]. Groups were matched for age, sex and body mass index in IBM^®^ SPSS^®^ version 27. Gait data were analysed with a custom Mathworks^®^ MatLab^®^ script and analysed in IBM^®^ SPSS^®^ version 27. The Shapiro–Wilk test indicated that normal distribution could not be assumed for a number of variables, consequently, data were compared using Kruskal–Wallis, then the Mann–Whitney test with Bonferroni correction (*α* = 0.05).

While gait analysis were the primary outcome of this study, patient reported outcomes were collected as a secondary outcome, measured at the time of treadmill assessment using the Oxford Knee Score (OKS) and EuroQol (EQ-5D) scores, Average scores were subject to the Mann–Whitney test according to the overall score and by individual question/domain (*α* = 0.05). CPKA subgroup data were descriptively analysed.

## Results

### Functional analysis

**Fig. 4:**
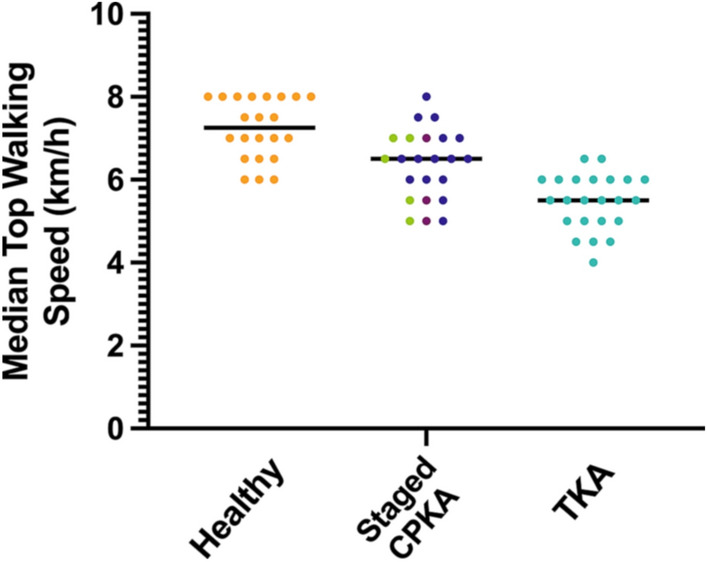
Median top walking speed (km/h) for subjects in study 4: staged combined partial knee arthroplasty (staged CPKA) compared to healthy subjects and posterior cruciate-retaining total knee arthroplasty (TKA). Of the staged CPKA group, the individual procedures are depicted: staged medial bicompartmental arthroplasty (green), staged lateral bicompartmental arthroplasty (light purple), staged bi-unicondylar arthroplasty (dark purple)

**Fig. 5:**
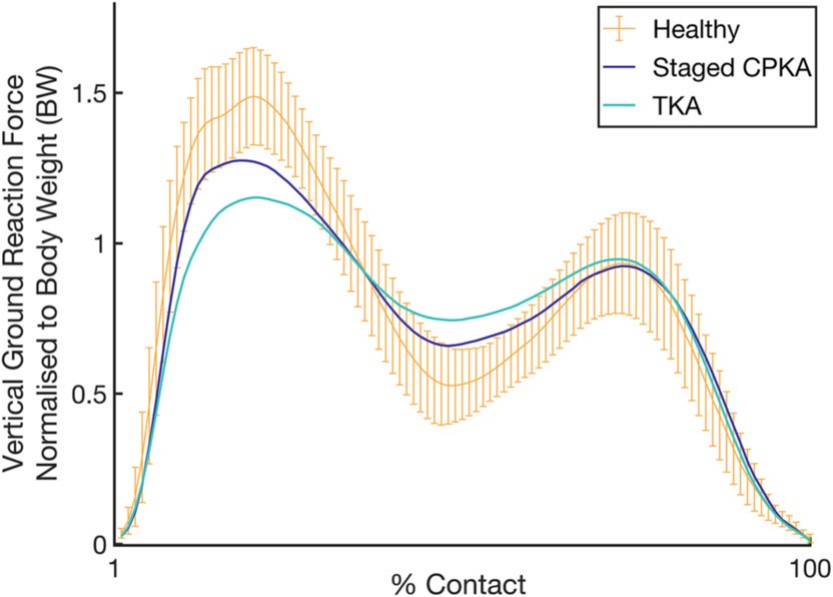
Vertical ground reaction forces during stance phase of gait for subjects revised from a partial to combined partial knee arthroplasty (CPKA) compared to primary posterior cruciate-retaining total knee arthroplasty (TKA), normal range for healthy subjects shown with 95% confidence intervals

The CPKA group walked, on average, 16% faster than primary TKA, though neither arthroplasty group walked as fast as the healthy subjects (*p* < 0.01, Fig. 4, Table 2.) This remained the case after correcting for leg length. At top walking speeds, CPKA had near-normal weight-acceptance rate (CPKA vs. healthy *p* = 1) whereas TKA weight-acceptance was reduced compared to healthy (*p* < 0.001) and CPKA (*p* < 0.006) subjects (Table 2, Fig. 5). Both implant groups demonstrated reduced maximum weight-acceptance force compared to the healthy subjects, though TKA was associated with a greater reduction (all < 0.01, Table 2, Fig. 5). Similarly, CPKA and TKA demonstrated higher mid-stance forces than healthy subjects (*p* < 0.001), but the difference was smaller following CPKA (*p* < 0.03). CPKA had nearer-normal contact time (CPKA vs. TKA *p* = 0.012) and double support time (CPKA vs. TKA *p* < 0.01).

**Fig. 6:**
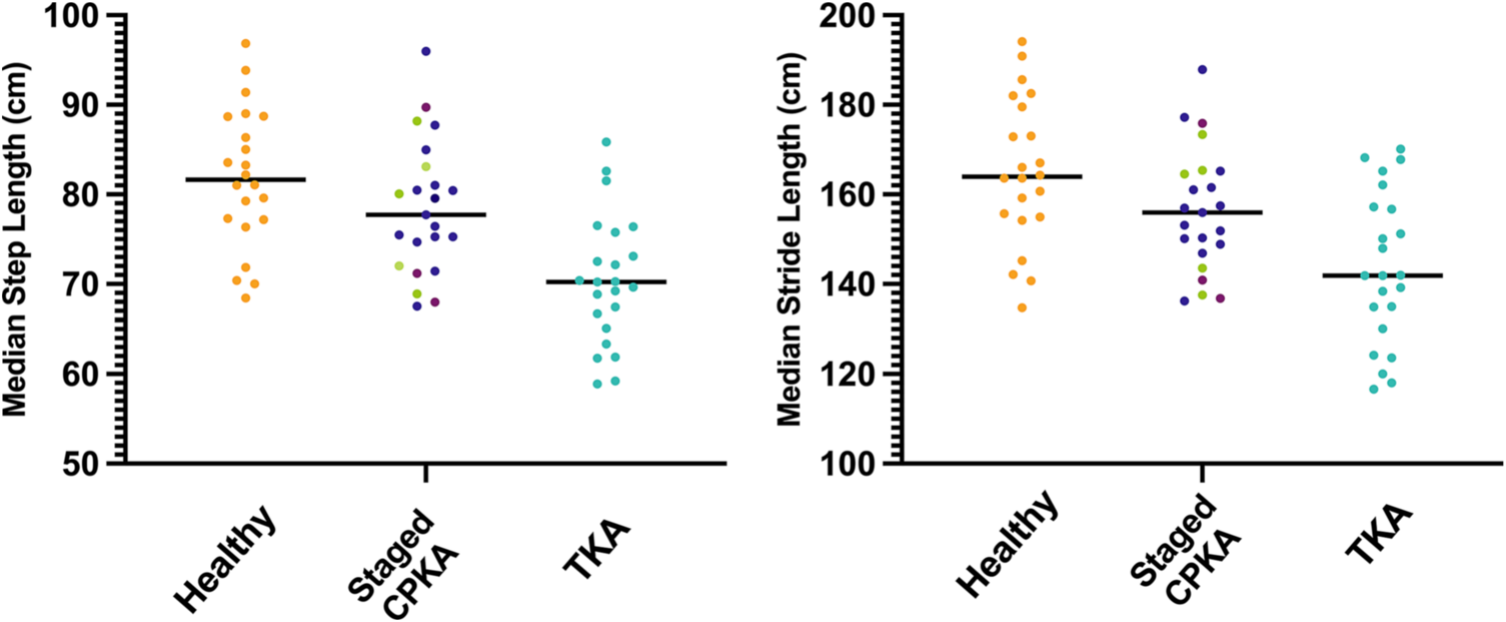
Median step length for all staged combined partial knee arthroplasty (staged CPKA) subjects compared to matched healthy subjects and posterior cruciate-retaining total knee arthroplasty subjects (TKA). Staged CPKA group, the individual procedures are depicted: staged medial bicompartmental arthroplasty (green), staged lateral bicompartmental arthroplasty (light purple), staged bi-unicondylar arthroplasty (dark purple)

Staged CPKA median step length were 78 cm (IQR 73–82 cm), 5% shorter than healthy controls, median 82 cm (IQR 77–88 cm), whilst TKA measured 15% shorter step lengths, median 70 cm (IQR 66–74 cm, Table 2, Fig. 6). Differences in step length were replicated in stride length: staged CPKA median stride length were 156 cm (IQR 148–165), 8 cm shorter than healthy subjects, median 164 cm (IQR 155–178 cm), but 12 cm longer than TKA, median 142 cm (IQR 133–157 cm, Table 2, Fig. 6).

### Patient reported outcomes

**Fig. 7:**
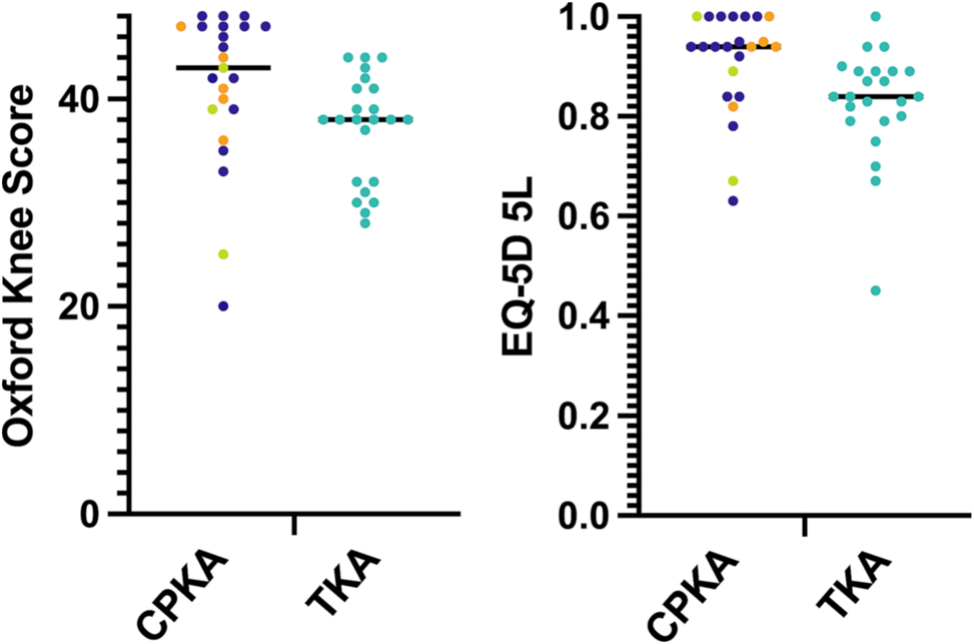
Patient reported outcome metrics for combined partial knee arthroplasty (CPKA) compared to posterior cruciate-retaining total knee arthroplasty (TKA). Left, Median Oxford Knee Score, where 48 represents best possible function. Right: Median EuroQol-5D 5L, where a score of 1 represents best possible quality of life. Each point represents one study subject. CPKA subgroups are shown: BCA-M (*n* = 5, orange), BCA-L (*n* = 3, green), Bi-UKA (*n* = 15, purple)

Mean OKS in the CPKA group was 41.3 ± 7.4 compared to TKA 37.1 ± 5.1 (*p* < 0.02, Fig. 7, Table 3). CPKA scored equal to or higher than TKA in all questions (Q) of the OKS, reaching significance in questions related to use of transport (Q3), kneeling (Q7) and instability symptoms (Q10, all *p* < 0.03). Similarly, CPKA subjects reported a mean EQ-5D 0.91 ± 0.1 compared to TKA 0.83 ± 0.2 (*p* = 0.006, Fig. 7, Table 4). CPKA scored equal or closer to 1 than TKA in all domains, reaching significance in mobility (*p* = 0.006), usual activities (*p* = 0.033) and pain (*p* = 0.033).

**Table 3:** Oxford Knee Scores between CPKA and TKA groups by overall score, where 48 is the best possible outcome, and by individual question

	CPKA	TKA	*p* value
Overall OKS	41.3 ± 7.4	37.1 ± 5.1	**0.02**
Q1. How would you describe the pain you usually have from your knee?	2.9 ± 1.0	2.7 ± 1.1	1
Q2. Have you had any trouble with washing and drying yourself (all over) because of your knee?	3.7 ± 0.6	3.8 ± 0.4	1
Q3. Have you had any trouble getting in or out of the car or using public transport because of your knee?	3.3 ± 0.9	2.7 ± 0.9	**0.027**
Q4. For how long have you been able to walk before pain from your knee becomes severe?	3.8 ± 0.6	3.6 ± 0.7	0.37
Q5. How painful has it been for you to stand up from a chair because of your knee?	3.3 ± 0.9	2.9 ± 0.8	0.11
Q6. Have you been limping when walking because of your knee?	3.4 ± 1.0	3.0 ± 1.1	0.46
Q7. Could you kneel down and get up again afterwards?	2.7 ± 1.3	1.8 ± 0.9	**0.02**
Q8. Have you been troubled by pain from your knee in bed at night?	3.5 ± 0.8	3.3 ± 0.8	1
Q9. How much has pain from your knee interfered with your usual work?	3.5 ± 0.9	3.6 ± 0.7	1
Q10. Have you felt that your knee may suddenly ‘give way’ or let you down?	3.8 ± 0.7	2.9 ± 0.8	**< 0.001**
Q11.Could you do the household shopping on your own?	3.7 ± 0.6	3.8 ± 0.5	1
Q12. Could you walk down one flight of stairs?	3.5 ± 0.7	3.1 ± 0.9	0.32

Each question has a maximum score of 4 for best outcome. Values shown are mean with standard deviation. Significant differences are highlighted in bold

**Table 4:** Euro-Qol 5D Scores between CPKA and TKA groups by overall score and by individual domain

	CPKA	TKA	*p* value
Overall EQ5D	0.91 ± 0.1	0.83 ± 0.1	**0.006**
Mobility	1.4 ± 0.7	2.1 ± 0.9	**0.006**
Self-care	1.2 ± 0.5	1.2 ± 0.4	1
Usual activities	1.5 ± 0.7	2.1 ± 0.9	**0.03**
Pain	1.7 ± 0.7	2.2 ± 0.7	**0.03**
Anxiety	1.2 ± 0.5	1.3 ± 0.6	1

The best possible outcome for the overall score is 1. Each domain is graded 1–5 where 1 is the best overall outcome. Values shown are mean with standard deviation. Significant differences are highlighted in bold

## Discussion

The most important finding of this study is that, despite second surgery, patients converted to CPKA, for native compartment osteoarthritis, were found to have a more normal gait than matched high functioning primary TKA subjects, in whom, surgery had been performed for one or two-compartment disease with the reverse trend not identified for any of the measured metrics. The marked differences in top walking speed are particularly relevant, since life expectancy improves with every 0.1 m/s increase [39]. During weight-acceptance and mid-stance, where the CPKA were significantly more normal than TKA, the quadriceps muscles are at their most active [30]. UKA and CPKA are significantly more anterior–posterior stable than TKA [15], and preserve the extensor efficiency of the knee, particularly at knee flexion angles associated with fast walking, whereas TKA is associated with a significant reduction in extensor efficiency of up to 43% at the same flexion angles [13]. These biomechanical differences may, in part, account for some of the observed differences in early stance.

Despite undergoing more than one operation, CPKA subjects reported good function on the OKS, corroborating the findings of others [34], with a good quality of life against EQ-5D. The OKS for TKA was in line with the TOPKAT study, a randomised control trial comparing primary UKA to TKA, suggesting that the TKA patients in this study performed as expected for a cohort of primary TKA [4]. However, while the overall scores were significantly different, the mean differences in OKS failed to exceed the “minimal important difference” threshold of five points [5] though the median score reached it. Although not validated for analysis by individual question, it is noteworthy that the largest differences were seen in the use of transport, kneeling and giving way, activities which rely heavily on the isokinetic quadriceps strength and extensor efficiency of the knee, where differences between CPKA and TKA are known to exist [13, 24, 11]. This ties in well with the gait data, since the most significant differences are seen in early stance, during weight acceptance and mid-stance, were the quadriceps are at their most active [30]. Whilst formal subgroup analysis is not appropriate due to demographic discrepancies, there is no obvious clustering of PROMs amongst the CPKA subgroups (Fig. 7).

Native compartment disease progression remains one of the most common reasons for revision of primary PKA, though the incidence varies between studies [10, 31, 33, 37]. In one series, of PFA revised to TKA, 56% resulted from disease progression in the tibiofemoral compartments [27], while in another, 76% of UKA were revised for the same reason [22]. However, the 20 year series from collected by Svard, reported just 2.3% revision rate for lateral compartment progression following UKA-M in the designer centre [36]. While at 7 years post-op, of 1000 patients whom underwent Phase 3 Oxford UKA-M, 2.5% of them required revision for progression of OA in the lateral compartment [33]. In 2020, the National Institute for Health and Clinical Excellence (NICE) revised their guidelines on knee replacement, for the first time, dividing revision surgery into ‘major’ and ‘minor’ procedures, noting they have different outcomes, despite being categorised together by joint registries. NICE also observed the thresholds for revision of PKA are lower than those for revision of primary TKA, in part due to the relative technical ease of PKA to TKA procedures [16].

There is good evidence that primary PKA is safer, more cost effective and higher functioning than primary TKA [4, 17, 21, 23, 40, 41]. Removal of a well-fixed PKA can result in significant bone loss, requiring increased constraint, augments, wedges and stems [6, 25, 33, 42]. The data on outcomes following revision of UKA to TKA are varied, with some studies reporting comparable rates of function and satisfaction to primary UKA [22, 29] while others report inferior outcomes compared to matched cohorts undergoing primary TKA [43]. Retention of a well-fixed, well-functioning primary PKA and targeted treatment of a newly degenerate compartment is an appealing option to PKA advocates, since the subsequent operation may be regarded as a second primary procedure. A designer series reported that the benefits of PKA also exist after staged Bi-UKA, with excellent functional outcomes, faster recovery, short hospital stays and very low complication rates [34]. This suggests that conversion to CPKA may be a good option in young people keen to avoid revision to TKA at an early age, and a safer alternative in older people or those considered high surgical risk. In terms of surgical invasiveness, the compartmental approach is a relatively minor undertaking compared to revision to TKA, and may, therefore be a conservative way of satisfying the NICE guidelines.

## Limitations

This paper is limited by the lack of pre-operative gait data for either the CPKA prior to revision or the primary TKA group which would be necessary to fully assess the change in function following either procedure. However, the purpose of this study was not to prove superiority of one treatment pathway over another, rather to provide a quantitative report and insight into the function of staged CPKA, and provide context for these data through comparison to a matched primary TKA patient group. The study were powered for gait analysis as a primary outcome, while PROMs were measured as a secondary outcome.

The status of the ACL in the TKA group was not documented prospectively, and has been inferred from the appearances of the pre-operative radiographs. Therefore, whilst all patients in the TKA group were potentially suitable for PKA or primary CPKA, there may have been reasons at the time why a TKA was considered more appropriate.

The route to CPKA for the subjects was variable and the inclusion of subgroups was necessary to power the study. The heterogeneity of the groups in terms of procedure order and type primary and secondary surgery, implant type, and time to second surgery is a limitation of the study, in as much as it is underpowered for specific implant combinations, brands or revision rates. A much larger study would be required to indicate whether some subtypes demonstrate advantages over others. Procedure subgroup analysis reported here is descriptive rather than statistical, due to small numbers and skewed sex demographics. Whilst we endeavoured to include all patients whom had undergone revision to CPKA under the senior author’s care, a significant number of patients were unsuitable, unable or unwilling to participate, which may have impacted upon the results. This study is underpowered to compare re-revision rates, though it is noted that four patients (5.4%) had undergone further ipsilateral knee arthroplasty surgery, excluding them from this study. The true re-revision rates for PKA revised to CPKA are unknown. The retrospective nature of this study meant that the timepoints in post-operative evaluations was highly variable, though the groups had a similar median follow-up time. TKA subjects with only single compartment OA were included, all of whom had undergone one procedure only, whilst all CPKA subjects had undergone two procedures and some had contralateral arthroplasty, all of which introduces bias in favour of TKA. Finally, all CPKA procedures were performed by an expert high-volume partial knee arthroplasty surgeon. There is evidence that surgeons with a higher proportion of PKA practice have better outcomes [32, 34], therefore the results may not be generalisable to low-volume PKA surgeons at this juncture, though this may be addressed in the future through additional training for arthroplasty surgeons or the use of assistive technologies including robotics, the latter having proved useful at restoring near-native alignment when used to implant primary Bi-UKA [3].

## Clinical relevance

Knee surgery is evolving, moving away from a “one definitive procedure” approach towards a patient-safety conscious, minor surgery “as required” strategy. This paper contributes toward the view that a compartmental approach to treatment of end-staged arthritis in a native compartment is high functioning and leads to good patient outcomes. It has the potential to support surgeons in their decision to perform primary PKA in the young, active patient, with reduced concern about subsequent surgery and the consequences of major revision. Further, it offers a safer, less invasive, bone-preserving alternative to revision to TKA in the event of progression of arthritis, which may prove particularly important in an ageing population or those who pose a high risk of medical complications.

## Conclusions

This study finds that a compartmental approach to native compartment degeneration following partial knee arthroplasty results in nearer-normal gait and improved patient satisfaction compared total knee arthroplasty.

## Supplementary information

Below is the link to the electronic supplementary material.

Supplementary file1 (DOCX 14 kb)

Supplementary file2 (DOCX 17 kb)
