# Supplementary material for: The compartmental approach to revision of partial knee arthroplasty results in nearer-normal gait and improved patient reported outcomes compared to total knee arthroplasty
Source: Knee Surg Sports Traumatol Arthrosc. 2021 Aug 20;31(3):1143–52. doi: 10.1007/s00167-021-06691-9 (PMC9957906; doi:10.1007/s00167-021-06691-9)
Supplement: Supplementary file 1 — Supplementary file1 (DOCX 14 kb) [file 167_2021_6691_MOESM1_ESM.docx]

| **Reason for Exclusion** | **N=** | **Details** | **Staged CPKA subgroup** |
| --- | --- | --- | --- |
| Further ipsilateral surgery | 1 | Medial Patella-femoral Ligament Reconstruction (RPKC class PR1) | BCA-M |
|  | 1 | Conversion to Tricompartmental Arthroplasty after 7yrs (RPKC class PR2b) | BCA-M |
|  | 1 | Revision to TKA after 6yrs (RPKC class PR3) | BCA-L |
|  | 1 | Revision of tibial component (mobile to fixed bearing) (RPKC class PR2b) | Bi-UKA |
|  | 1 | Conversion to Tricompartmental Arthroplasty after 2yrs (RPKC class PR2b) | Bi-UKA |
|  | 1 | Ipsilateral foot surgery | Bi-UKA |
| Previous significant trauma | 1 | Femoral intramedullary nail in situ | BCA-M |
|  | 4 | Lateral tibial plateau fracture prior to UKA-L | Bi-UKA |
| Medically Unfit | 2 | Recent cardiac surgery | * |
|  | 3 | Significant cardiac disease | * |
|  | 1 | Significant pulmonary disease | * |
|  | 1 | Recent kidney transplant | * |
|  | 1 | Significant spinal surgery | * |
| Unable to consent | 1 | Dementia | * |

Supplementary Table A. Reasons for exclusion of CPKA subjects from the study. *concealed to prevent individual patient identification
