# Supplementary material for: The compartmental approach to revision of partial knee arthroplasty results in nearer-normal gait and improved patient reported outcomes compared to total knee arthroplasty
Source: Knee Surg Sports Traumatol Arthrosc. 2021 Aug 20;31(3):1143–52. doi: 10.1007/s00167-021-06691-9 (PMC9957906; doi:10.1007/s00167-021-06691-9)
Supplement: Supplementary file 2 — Supplementary file2 (DOCX 17 kb) [file 167_2021_6691_MOESM2_ESM.docx]

| Subject | Subgroup | Primary Procedure | Implant | Manufacturer | Second Procedure | Implant | Manufacturer |
| --- | --- | --- | --- | --- | --- | --- | --- |
| 1 | Bi-UKA | UKA-M | Oxford® Partial Knee | Zimmer Biomet | UKA-L | Oxford® Domed Lateral | Zimmer Biomet |
| 2 | Bi-UKA | UKA-M | Oxford® Partial Knee | Zimmer Biomet | UKA-L | Oxford® Domed Lateral | Zimmer Biomet |
| 3 | Bi-UKA | UKA-M | Oxford® Partial Knee | Zimmer Biomet | UKA-L | Oxford® Domed Lateral | Zimmer Biomet |
| 4 | Bi-UKA | UKA-M | Oxford® Partial Knee | Zimmer Biomet | UKA-L | Oxford® Fixed Lateral | Zimmer Biomet |
| 5 | Bi-UKA | UKA-M | Oxford® Partial Knee | Zimmer Biomet | UKA-L | Oxford® Fixed Lateral | Zimmer Biomet |
| 6 | Bi-UKA | UKA-M | Oxford® Partial Knee | Zimmer Biomet | UKA-L | Oxford® Fixed Lateral | Zimmer Biomet |
| 7 | Bi-UKA | UKA-M | Oxford® Partial Knee | Zimmer Biomet | UKA-L | Oxford® Fixed Lateral | Zimmer Biomet |
| 8 | Bi-UKA | UKA-M | Oxford® Partial Knee | Zimmer Biomet | UKA-L | Oxford® Fixed Lateral | Zimmer Biomet |
| 9 | Bi-UKA | UKA-L | Oxford® Domed Lateral | Zimmer Biomet | UKA-M | Oxford® Partial Knee | Zimmer Biomet |
| 10 | Bi-UKA | UKA-L | Oxford® Domed Lateral | Zimmer Biomet | UKA-M | Oxford® Partial Knee | Zimmer Biomet |
| 11 | Bi-UKA | UKA-L | Oxford® Domed Lateral | Zimmer Biomet | UKA-M | Oxford® Partial Knee | Zimmer Biomet |
| 12 | Bi-UKA | UKA-L | Oxford® Domed Lateral | Zimmer Biomet | UKA-M | Oxford® Partial Knee | Zimmer Biomet |
| 13 | Bi-UKA | UKA-L | Oxford® Domed Lateral | Zimmer Biomet | UKA-M | Oxford® Partial Knee | Zimmer Biomet |
| 14 | Bi-UKA | UKA-L | JOURNEY™ UNI | Smith & Nephew | UKA-M | Oxford® Partial Knee | Zimmer Biomet |
| 15 | Bi-UKA | UKA-L | St Georg SLED | LINK® | UKA-M | St Georg SLED | LINK® |
| 16 | BCA-M | UKA-M | Oxford® Partial Knee | Zimmer Biomet | PFA | Gender Solutions® | Zimmer Biomet |
| 17 | BCA-M | UKA-M | Oxford® Partial Knee | Zimmer Biomet | PFA | JOURNEY™ PFJ | Smith & Nephew |
| 18 | BCA-M | UKA-M | Oxford® Partial Knee | Zimmer Biomet | PFA | Avon™ | Stryker® |
| 19 | BCA-M | PFA | JOURNEY™ PFJ | Smith & Nephew | UKA-M | JOURNEY™ UNI | Smith & Nephew |
| 20 | BCA-M | PFA | KineMatch® PFR | Kinamed® incorporated | UKA-M | Oxford® Partial Knee | Zimmer Biomet |
| 21 | BCA-L | UKA-L | Oxford® Fixed Lateral | Zimmer Biomet | PFA | SIGMA® HP | DePuy Synthes |
| 22 | BCA-L | UKA-L | Oxford® Domed Lateral | Zimmer Biomet | PFA | P.F.C® SIGMA® | DePuy Synthes |
| 23 | BCA-L | UKA-L | Uniglide™ | Corin | PFA | JOURNEY™ PFJ | Smith & Nephew |

Supplementary Table B. Implant order and combinations by brand and manufacturer for subjects in the study.
